# Avian malaria on Madagascar: bird hosts and putative vector mosquitoes of different *Plasmodium* lineages

**DOI:** 10.1186/s13071-016-1939-x

**Published:** 2017-01-05

**Authors:** Sandrine Schmid, Anke Dinkel, Ute Mackenstedt, Michaël Luciano Tantely, Fano José Randrianambinintsoa, Sébastien Boyer, Friederike Woog

**Affiliations:** 1 Universität Hohenheim, Institut für Zoologie, FG Parasitologie, Emil-Wolff-Straße 34, 70593 Stuttgart, Germany; 2 Institut Pasteur de Madagascar, Unité d’Entomologie Médicale, BP 1274 Avaradoha, Antananarivo 101, Madagascar; 3 Université de Reims, 9 Boulevard de la Paix, 51100 Reims, France; 4 Staatliches Museum für Naturkunde,Ornithology, Rosenstein1, 70191 Stuttgart, Germany

**Keywords:** Haemosporida, *Uranotaenia*, *Anopheles mascarensis*, *Haemoproteus*, Ploceidae, Pycnonotidae

## Abstract

**Background:**

Avian malaria occurs almost worldwide and is caused by Haemosporida parasites (*Plasmodium*, *Haemoproteus* and *Leucocytozoon*). Vectors such as mosquitoes, hippoboscid flies or biting midges are required for the transmission of these parasites. There are few studies about avian malaria parasites on Madagascar but none about suitable vectors.

**Methods:**

To identify vectors of avian *Plasmodium* parasites on Madagascar, we examined head, thorax and abdomen of 418 mosquitoes from at least 18 species using a nested PCR method to amplify a 524 bp fragment of the haemosporidian mitochondrial cytochrome *b* gene. Sequences obtained were then compared with a large dataset of haemosporidian sequences detected in 45 different bird species (*n* = 686) from the same area in the Maromizaha rainforest.

**Results:**

Twenty-one mosquitoes tested positive for avian malaria parasites. *Haemoproteus* DNA was found in nine mosquitoes (2.15%) while *Plasmodium* DNA was found in 12 mosquitoes (2.87%). Seven distinct lineages were identified among the *Plasmodium* DNA samples. Some lineages were also found in the examined bird samples: *Plasmodium* sp. WA46 (EU810628.1) in the Madagascar bulbul, *Plasmodium* sp. mosquito 132 (AB308050.1) in 15 bird species belonging to eight families, *Plasmodium* sp. PV12 (GQ150194.1) in eleven bird species belonging to eight families and *Plasmodium* sp. P31 (DQ839060.1) was found in three weaver bird species.

**Conclusion:**

This study provides the first insight into avian malaria transmission in the Maromizaha rainforest in eastern Madagascar. Five *Haemoproteus* lineages and seven *Plasmodium* lineages were detected in the examined mosquitoes. Complete life-cycles for the specialist lineages WA46 and P31 and for the generalist lineages mosquito132 and PV12 of *Plasmodium* are proposed. In addition, we have identified for the first time *Anopheles mascarensis* and *Uranotaenia* spp. as vectors for avian malaria and offer the first description of vector mosquitoes for avian malaria in Madagascar.

## Background

The island of Madagascar is located approximately 400 km east of Africa in the Indian Ocean. Due to its isolation from mainland India and Africa it has many endemic species and is classified as an important biodiversity hotspot [1]. More than half of Madagascar′s breeding birds are endemic. This makes Madagascar an interesting area for the examination of the ecological and evolutionary dynamics of vector-host-parasite associations of avian malaria.

Avian malaria is caused by haemosporidian parasites including the genera *Plasmodium*, *Haemoproteus* and *Leucocytozoon*. In contrast to human malaria, avian malaria is distributed almost worldwide and has been detected in a wide range of species [2]. The three genera have similar life-cycles with differences during the asexual phase in the peripheral blood of their host [3]. The life-cycle involves the sexual phase and sporogony both occurring in the invertebrate host (vector) and the merogony and development of gametocytes which takes place in avian hosts [4]. For natural transmission of *Plasmodium* to the vertebrate host, the parasite undergoes a series of obligatory developmental, propagative and migrational processes inside a mosquito vector. These include zygote formation and ookinete development in the midgut lumen, oocyst formation and sporogony on the basal side of the midgut, sporozoite migration through the haemocoel, and invasion of the salivary glands [5]. Sporozoites, the infective stages, are then present in the salivary gland (thoracic part) of a mosquito and detectable for several weeks [6]. Since the end of the 20th Century, a number of field and laboratory experimental infection studies were used to identify dipterans as vectors transmitting haemosporidian parasites (e.g. [7, 8]). Recently, most studies use molecular methods to identify vector feeding preferences [9] and sporogonic stages of the parasites in salivary glands to identify potential vectors [10].

To date, few studies exist on blood parasites of Malagasy birds. Either blood samples were examined microscopically [11, 12] or just a small number was analyzed by PCR [13]. So far, data about vectors in Madagascar transmitting avian malaria parasites are lacking. To identify mosquitoes as suitable vectors for *Plasmodium* species, a demonstration of infective sporozoites in insects is essential. Due to predominant light infections and low parasite prevalence (often < 1%) in the majority of vector populations, the applicability of insect dissection and microscopic examination methods is limited in wildlife [6]. For parasite detection we therefore used a highly sensitive nested PCR. Positive results allowed us to determine significant links between mosquitoes and haemosporidian species.

The purpose of our study was to assess the presence of avian malaria parasites in potential mosquito vectors and avian hosts using molecular techniques and to estimate infection rates among mosquito populations in the Maromizaha rainforest located in eastern Madagascar (Andasibe). In addition, *Plasmodium* spp. sequences isolated from mosquitoes were compared to those found in birds within the study area. A complete life-cycle with putative vector, host and parasite is proposed.

## Methods

Mosquitoes and birds were caught in the Maromizaha rainforest located in the eastern part of Madagascar (18°56′49″S, 48°27′33″E), 30 km from Moramanga city, with an altitude above 943 m and a highest peak of 1,213 m. The area is protected and part of the Mantadia-Maromizaha-Zahamena rainforest corridor, a terrain that consists of hills with mountain ridges, valleys and small streams, dense and humid evergreen forest covers.

A total of 418 adult mosquitoes of at least 18 species (Table 1) were collected using twelve CDC (Center for Disease Control and Prevention) light traps [14] during one week (November 17–21, 2014). The sampling period was based on the knowledge of high mosquito diversity and density in forested areas of the central highland at the beginning of the rainy season (i.e. November) [15]. The traps were operated for 12 h periods from 6 pm to 6 am at each sampling point. To sample a representative mosquito population, traps were distributed along three sampling lines, thus, valley-floor, slope and ridge for each biotope, with four traps in each sampling point. Mosquitoes were anesthetized with chloroform vapor and identified using the keys of Ravaonjanahary [16] for *Aedes*, Grjebine [17] for *Anopheles*, Doucet [18] for *Coquillettidia*, Edwards [19] for *Culex*, Brunhes & Hervy [20] for *Orthopodomyia* and da Cunha Ramos [21] for *Uranotaenia*. After drying, mosquitoes were stored in ELISA plates to allow identification. DNA extractions were performed separately from head, thorax and abdomen of each mosquito in order to determine the infection rates and diversity of parasite lineages within the thoracic (including salivary glands) and abdominal parts, using the Zymo Research extraction kit (Quick-gDNA™ MiniPrep; Zymo Research Europe GmbH, Freiburg, Germany). The isolated DNA was stored at -20 °C until used.

**Table 1:** PCR results of examined mosquito species

Genus	Species	*n*	H/T	A	*Plasmodium*	*Haemoproteus*
*Aedes*	*circumluteolus*	9				
*Anopheles*	*lacani*	1				
	*mascarensis*	2	1		1	
	*gambiae* (*s.l*.)	3				
*Coquillettidia*	*grandidieri*	4				
*Culex*	*annulioris*	5		1		1
	*antennatus*	3				
	*decens*	11				
	*giganteus*	8				
	*pipiens*	164	3	3	2	4
	sp.	5				
*Lutzia*	*tigripes*	3				
*Orthopodomyia*	*milloti*	1				
*Uranotaenia*	*alboabdominalis*	45	3		2	1
	*anopheloides*	4				
	n. sp.^*^	96	5	2	4	3
	*neireti*	6	1		1	
	sp.	48	1	1	2	
		418	14	7	12 (2.87%)	9 (2.15%)

*Abbreviations*: *n* number of different individuals screened using PCR, *H/T* positive head or thorax samples, *A* positive abdomen samples

^*^ A new species in process of being described (Tantely, pers. Comm.)

A total of 686 blood samples were collected from 45 bird species mist-netted in the Maromizaha rainforest in the years 2006, 2007, 2010, 2012 and 2014. Sampling was done by taking a drop of blood from the brachial vein and stored in buffer [22]. For DNA extraction we used QIAamp DNA Blood Mini Kit (QIAGEN, Hilden, Germany) following the manufacturer's instructions. DNA was stored at -20 °C until further use.

To prevent contamination with target DNA, a negative control was included in each test run as well as a positive control to ensure PCR was working properly. Target sequence for amplification was a part of the haemosporidian mitochondrial cytochrome *b* gene. PCR was conducted in two steps whereby the primer pair HAEMNF and HAEMNR2 [23] was used to amplify a 580 bp fragment in the first PCR, and the internal primer pair HAEMF and HAEMR2 [24] used to amplify a 524 bp fragment in the second PCR. The reaction mixture consisted of 10 mM Tris-HCl, 50 mM KCl, 2 mM MgCl_2_, 20 pmol of each primer, 200 μM of each dNTP, 1.25 units Ampli-Taq (Applied Biosystems, Carlsbad, USA) and for the first PCR approximately 10–100 ng DNA in a total volume of 50 μl. For the second PCR 2 μl of the amplification product was used as template. Both PCRs were performed for 40 cycles with each cycle consisting of denaturation at 94 °C for 30 s, annealing at 50 °C for 60 s and elongation at 72 °C for 45 s. After amplification, 5 μl of the PCR products stained with GelRed™ (BIOTREND, Köln, Germany) were viewed on a 1.5% agarose gel. Amplification products were then purified using the PCR Product Purification Kit (Roche, Mannheim, Germany) and after sequencing (GATC Biotech AG) the resulting sequences were compared to sequences published in GenBank [25].

## Results

A total of 418 mosquitoes were examined, representing at least 18 species of seven genera (Table 1). *Culex* (46.9%) and *Uranotaenia* (47.6%) were the two most frequent mosquito genera collected in the study. Other mosquito species found include those of the genera *Aedes*, *Anopheles*, *Coquillettidia*, *Lutzia* and *Orthopodomyia*. Using the two step PCR, we found 21 of the 418 mosquitoes (5.02%) to be haemosporidian DNA-positive.

These positive samples were of the mosquito species *Anopheles mascarensis*, *Culex annulioris*, *C. pipiens*, *Uranotaenia alboabdominalis*, *Uranotaenia neireti*, *Uranotaenia* n. sp. (Tantely, pers. Comm.) and *Uranotaenia* sp. The haemosporidian DNA detected include five *Haemoproteus* lineages, isolated from nine mosquitoes and seven *Plasmodium* lineages from 12 mosquitoes (Table 2).

**Table 2:** Isolated parasite lineages of this study

Parasite lineage	Homology (%) [GenBank ID]	Mosquito vector	Bird host species
*Haemoproteus* spp.			
*Ual1*		*Uranotaenia alboabdominalis*	
*Cp1*	99 [AY099042.1]	*Culex pipiens*	
*Cp2*	99 [AY099042.1]	*Culex pipiens* (*n* = 3); *Culex annulioris*	
*Uan1*	98 [AY099040.1]	*Uranotaenia* n. sp.	*Nesillas typica* (*n* = 1)
*Haplotype* 1252	100 [KJ488706.1]	*Uranotaenia* n. sp. (*n* = 2)	
*Plasmodium* spp.			
*Ual1*	95 [EU834703.1]	*Uranotaenia alboabdominalis* (T)	
*Uan1*	97 [JN819332.1]	*Uranotaenia* n. sp. (T)	
*Cp1*	99 [AB308050.1]	*Culex pipiens* (T)	
*WA46*	100 [EU810628.1]	*Uranotaenia sp.* (T + A); *Ur. alboabdominalis* (T); *Ur. neireti* (T)	*Hypsipetes madagascariensis* (*n* = 5)
*Mosquito* 132	100 [AB308050.1]	*Culex pipiens* (T); *Uranotaenia* n. sp. (T + A)	51 birds of 15 species
*PV12*	100 [GQ150194.1]	*Anopheles mascarensis* (T)	37 birds of 11 species
*P31*	100 [DQ839060.1]	*Uranotaenia* n. sp. (T)	39 birds of the family Ploceidae

*Abbreviations*: *T* thoracic part, *A* abdominal part

Only mosquitoes with positive thoracic parts are considered to be putative vectors since infectious sporozoites are exclusively present in the salivary glands of arthropod vectors. The resulting haemosporidian DNA sequences of mosquitoes with positive thoracic parts therefore include *Plasmodium* sp. Uan1 (*n =* 1; from *Uranotaenia* n. sp.); *Plasmodium* sp. WA46 (EU810628.1) (*n =* 3; from *Uranotaenia* sp., *Ur. neireti* and *Ur. alboabdominalis*); *Plasmodium* sp. Ual1 (*n =* 1; from *Ur. alboabdominalis*); *Plasmodium* sp. Cp1 (*n =* 1; from *Culex pipiens*); *Plasmodium* sp. mosquito132 (AB308050.1) (*n =* 2; from *Culex pipiens, Uranotaenia* n. sp.); *Plasmodium* sp. PV12 (GQ150194.1) (*n =* 1; from *Anopheles mascarensis*); and *Plasmodium* sp. P31 (DQ659572.1) (*n =* 1; from *Uranotaenia* n. sp.). The sequences were compared to those found in bird blood samples collected in the years 2006, 2007, 2010, 2012 and 2014.

The *Plasmodium* sp. mosquito132 lineage was found in 51 bird blood samples from 15 bird species (*Foudia omissa*, *Xanthomixis cinereiceps*, *Nesillas typica*, *Hypsipetes madagascariensis*, *Copsychus albospecularis*, *Zosterops maderaspatanus*, *Monticola sharpei*, *Ploceus nelicourvi*, *Foudia madagascariensis*, *Xanthomixis zosterops*, *Saxicola torquatus*, *Tylas eduardi*, *Philepitta castanea* and *Pseudobias wardi*) belonging to eight bird families (Acrocephalidae, Bernieridae, Muscicapidae, Philepittidae, Ploceidae, Pycnonotidae, Vangidae and Zosteropidae).

*Plasmodium* sp. P31 was also found in 39 blood samples of weaver birds (Ploceidae), *Foudia omissa* (*n* = 35), *Foudia madagascariensis* (*n* = 3) and *Ploceus nelicourvi* (*n* = 1). The lineage *Plasmodium* sp. PV12 was found in 37 blood samples of 11 bird species (*Foudia omissa*, *Saxicola torquatus*, *Philepitta castanea*, *Ploceus nelicourvi*, *Nesillas typica*, *Bernieria madagascariensis*, *Hypsipetes madagascariensis*, *Phedina borbonica*, *Foudia madagascariensis*, *Copsychus albospecularis*, *Motacilla flaviventris*) belonging to eight bird families (Acrocephalidae, Bernieridae, Hirundinidae, Motacillidae, Muscicapidae, Philepittidae, Ploceidae and Pycnonotidae).

*Plasmodium* sp. WA46 was as well found in five Madagascar Bulbul (*Hypsipetes madagascariensis*, Pycnonotidae) blood samples.

## Discussion

Blood-sucking arthropods are necessary for the life-cycle of avian malaria parasites. They act as definitive hosts and vectors of avian malaria parasites and are thus responsible for the transmission. Identification of such vector arthropods is therefore essential to unravel the transmission cycles of vector borne diseases like avian malaria [26]. For many parasite species of the genera *Plasmodium*, *Haemoproteus* or *Leucocytozoon* suitable arthropod vectors have already been identified [27]. Mosquitoes are the only known vectors for *Plasmodium* species.

To identify vectors of avian malaria on Madagascar, we examined 418 mosquitoes from at least 18 species individually using a part of the mitochondrial cytochrome *b* gene. Sequences found in the mosquitoes were compared to a large dataset of sequences isolated from 45 bird species (*n* = 686) of the same area. Twenty-one mosquitoes were found to contain DNA of avian Haemosporida.

We found *Haemoproteus* DNA in nine mosquitoes. *Haemoproteus* species develop oocysts in the head, thorax and midgut wall of mosquitoes but undergo abortive sporogonic development [6]. Therefore, mosquitoes are not competent vectors for this parasite. Haemosporidian parasites can be used to determine links between blood-sucking insects and their blood-source animals [6]. All identified *Haemoproteus* lineages in the present study were similar or highly homologous to sequences previously isolated from birds. This evidence therefore implicates that *Uranotaenia alboabdominalis*, *Uranotaenia* n. sp.*, Culex annulioris* and *Culex pipiens* feed on birds in the study area and could be possible vectors for *Plasmodium* species.

Seven *Plasmodium* lineages were detected in 12 mosquito species. Of all examined mosquito species in this study containing *Plasmodium* DNA, only *Culex pipiens* has been previously known to be a suitable vector for avian *Plasmodium* species [27]. *Culex pipiens* was the most abundant mosquito in our study (39%) followed by the newly-described species *Uranotaenia* n. sp. (22.7%)*.* We detected DNA of *Plasmodium* only in three *Culex pipiens* samples (2× thorax, 1× abdomen), indicating that this mosquito species may not play the most important role for avian *Plasmodium* transmission in the study area.

The endemic mosquito species *Anopheles mascarensis* is reported to act as a vector of *Plasmodium falciparum*, the causative agent of human malaria [28]. We found an avian *Plasmodium* lineage in the thorax of one sampled *Anopheles mascarensis* indicating that this endemic mosquito species plays an important role for both, human and avian malaria transmission.

Ten of the 12 (83.3%) *Plasmodium* lineages found were isolated from mosquitoes belonging to the genus *Uranotaenia*. This is the first report of *Uranotaenia* species acting as vectors for avian malaria. Although further studies are needed, the genus *Uranotaenia* might even be the major vector for avian malaria in this area.

The isolated *Plasmodium* lineages were compared to previously published sequences in GenBank and thus identified. We isolated the sequence *Plasmodium* sp. Ual1 from the thorax of one *Uranotaenia alboabdominalis*. The sequence was 95% homologous to the previously described lineage *Plasmodium minuoviride* haplotype CCA0640 (EU834703.1). This lineage was isolated by Perkins et al. [29] from *Prasinohaema prehensicauda*, a skink (Squamata) endemic to Papua New Guinea. Because species of the genus *Uranotaenia* are known to feed on cold-blooded animals [15] and due to the high homology we conclude that the sequence we found also stems from a *Plasmodium* species infecting reptiles. The lineages *Plasmodium* sp. Uan1 (isolated from thorax of *Uranotaenia* n. sp.) and *Plasmodium* sp. Cp1 (isolated from thorax of *Culex pipiens*) were highly homologous to sequences previously isolated from birds. Therefore, we conclude that these sequences represent two new *Plasmodium* lineages infecting birds. The other four sequences isolated were identical with lineages previously described and were also found in bird blood samples examined during this study. The presence of the identical haemosporidian cytochrome *b* sequence in birds and mosquitoes could be an indication of a vector-host relationship between the species described.

We isolated the lineage *Plasmodium* sp. WA46 [30] from four mosquitoes belonging to the genus *Uranotaenia*. The parasite sequence was found in the thoracic parts of *Uranotaenia alboabdominalis*, *Ur. neireti* and in one undescribed *Uranotaenia* species. We conclude that all three mosquito species are probably suitable vectors for the *Plasmodium* lineage WA46. The very same lineage was also found in five bird blood samples of the Madagascar bulbul (*Hypsipetes madagascariensis*, Pycnonotidae). The blood was sampled from different individuals in the years 2007, 2010, 2012 and 2014 suggesting a stable occurrence in the study area. The lineage *Plasmodium* sp. WA46 was originally isolated by Beadell et al. [30] from the common bulbul (*Pycnonotus barbatus*, Pycnonotidae). Our findings support the hypothesis, that the lineage WA46 is a specialist for the bird family Pycnonotidae. As *Hypsipetes madagascariensis* is the only bulbul species on Madagascar we assume that the life-cycle of *Plasmodium* sp. WA46 in our study area includes at least three vector mosquito species of the genus *Uranotaenia* and the host bird *Hypsipetes madagascariensis*.

The lineage *Plasmodium* sp. mosquito132 [26] was isolated from the thoracic parts of *Culex pipiens* and *Uranotaenia* n. sp. in this study. Ejiri et al. [26] originally isolated the lineage from *Culex pipiens quinquefasciatus* captured in Japan. We supported the role of *Culex pipiens* as vector for this lineage also on Madagascar but with *Uranotaenia* n. sp. we found a second vector mosquito. We isolated DNA of *Plasmodium* sp. mosquito132 also from 51 bird blood samples. The samples stem from individuals of 15 bird species belonging to eight bird families. The finding of the lineage in so many diverse bird species leads us to the conclusion that *Plasmodium* sp. mosquito132 is a generalist.

From the endemic mosquito *Anopheles mascarensis* we isolated the lineage *Plasmodium* sp. PV12 [31]. The lineage was originally isolated from the mosquito *Coquillettidia aurites* in Cameroon. We did not find the lineage in the four samples of *Coquillettidia grandidieri* examined in this study. Because of the small sample size we cannot exclude this mosquito species as a possible vector. Our findings suggest that *Anopheles mascarensis* is a vector of *Plasmodium* sp. PV12 in the study area. We also detected this lineage in 37 bird blood samples. The samples are from eleven 11 bird species belonging to eight families. The finding of the lineage in so many diverse bird species leads us to the conclusion that *Plasmodium* sp*.* PV12 is a generalist.

The *Plasmodium* lineage isolated from *Uranotaenia* n. sp. was identical to the previously described *Plasmodium* sp. P31 [32]. The lineage was originally isolated from *Foudia madagascariensis* (Ploceidae) captured on Madagascar. In the current study the same sequence was isolated from 39 blood samples of three weaver bird species, including *Foudia omissa*, *Foudia madagascariensis* and *Ploceus nelicourvi*. Therefore, *Plasmodium* sp. P31 seems to be specific to weaver birds (Ploceidae). Fodies were among the most frequently sampled birds. From observational studies they are known to move from the forest to the open, more degraded areas, and may therefore spread blood parasites into more pristine areas. The weaver bird lineage was detected in 39 samples from three different years (2006, 2007, 2010, 2012 and 2014). The high and apparently stable prevalence in the birds suggests that there is a stable life-cycle of *Plasmodium* sp. P31 on Madagascar with its intermediate host being weaver birds and *Uranotaenia* n. sp. as the first described vector.

To confirm the role of the genus *Uranotaenia*, *Anopheles mascarensis* and *Culex pipiens* in the transmission cycle of the reported avian malaria parasites on Madagascar, further analyses of blood-fed mosquitoes from infection experiments are required.

## Conclusions

This study provides the first insight into avian malaria transmission in the Maromizaha forest in eastern Madagascar. Five *Haemoproteus* lineages and seven *Plasmodium* lineages were detected in the examined mosquitoes. Complete life-cycles for the specialist *Plasmodium* lineages WA46 and P31 and for the generalist *Plasmodium* lineages mosquito132 and PV12 are proposed. *Plasmodium* sp. WA46 is transmitted from mosquitoes of the genus *Uranotaenia* to birds belonging to the family Pycnonotidae. *Plasmodium* sp. P31 is transmitted from *Uranotaenia* n. sp. to birds belonging to the family Ploceidae. *Plasmodium* sp. mosquito132 is transmitted from *Culex pipiens* and *Uranotaenia* n. sp. to at least 15 different bird species on Madagascar. *Plasmodium* sp. PV12 is transmitted from *Anopheles mascarensis* to at least 11 different bird species on Madagascar.

This study identified *Anopheles mascarensis* and *Uranotaenia* spp. as putative vectors for avian malaria for the first time and offers the first description of vector mosquitoes for avian malaria on Madagascar.
